# First episode psychosis in a young person with a diagnosis of Autistic Spectrum Disorder: A Case report

**DOI:** 10.1192/j.eurpsy.2024.946

**Published:** 2024-08-27

**Authors:** P. Raghvani, C. Inogbo, V. Damle

**Affiliations:** Hope Unit, Pennine Care NHS Foundation Trust, Bury, Manchester, United Kingdom

## Abstract

**Introduction:**

Psychotic disorders are significant comorbidities in young people with Autistic Spectrum Disorder (ASD). Evidence suggests that ASD & psychosis present with overlapping clinical features & cognitive symptoms leading to misdiagnosis (Trevisan *et al.* Front.Psych 2020;11:548). Clinicians encounter diagnostic dilemma during assessment of psychosis in adolescents with ASD.

**Objectives:**

To discuss the clinical challenges in the assessment & treatment of young people with ASD & comorbid psychosis.

**Methods:**

A case report of a young girl with ASD & comorbid psychotic illness.

**Results:**

A young girl with ASD was admitted to CAMHS inpatient Unit with unusual beliefs & perceptual disturbances. She reported hearing the voice of ‘Hydrogis’ who was talking to her about his girlfriend. She made a voodoo doll & tried to set it on fire, as she believed that this would kill the girlfriend. She also heard voices of characters from a TV show, discussing her in third person. She absconded from home due to the distress associated. She attempted suicide by tying a ligature. She was seen responding to external stimuli, laughing incongruously & was thought disordered. Despite never being to USA, she spoke in American accent. She lacked insight & struggled to differentiate reality from fantasy. The aim of admission was to determine if the symptoms were part of ASD or a psychotic disorder. She had medication free assessment but continued to be very distressed. We commenced Aripiprazole which was optimised. She responded well to the treatment & was discharged to the care of Early Intervention in Psychosis team with partial remission of symptoms.

**Conclusions:**

Historically psychotic illnesses & ASD were thought to be closely linked. Research suggest that they are two separate disorders with specific onset, progress, signs & symptoms. ASD might be misdiagnosed as psychosis as difficulties in communication may resemble thought disorder, ‘melt down’ may mimic catatonia & difficulties in recognising others’ intentions may mimic paranoia. Our patient was experiencing first episode psychosis in late adolescence. This age of onset is consistent with research findings. A study to differentiate between ASD & psychosis found that positive symptoms like hallucinations & delusions were suggestive of psychosis while odd emotional gestures, stereotyped speech & restricted interests indicated ASD. Our patient predominantly had positive symptoms of delusions, hallucinations & thought disorder, hence our diagnosis of psychotic episode. In some cases, it is difficult to differentiate childhood fantasies from delusional beliefs (Ribolsi *et al.* Front.Psych 2022;13:768586). Bleuler explains that children with ASD replace imperfect realities with imaginations & hallucinations but Michael Rutter claims that autistic children lack fantasy. There are varying views on this subject & this is the challenge we faced when treating this young person.

**Disclosure of Interest:**

None Declared

